# Updated resource of 180K soybean SNP genotyping array based on the T2T reference genome

**DOI:** 10.1371/journal.pone.0335227

**Published:** 2025-12-05

**Authors:** Ji-Hun Hwang, Sungwoo Lee, Ju Seok Lee, Kyung Do Kim

**Affiliations:** 1 Department of Integrative Biological Sciences and Industry, Sejong University, Seoul, Republic of Korea; 2 Department of Crop Science, Chungnam National University, Daejeon, Republic of Korea; 3 Bio-Evaluation Center, Korea Research Institute of Bioscience and Biotechnology, Cheongju, Republic of Korea; 4 Institute for Advanced Plant Breeding and Phytochemicals (IAPBP), Sejong University, Seoul, Republic of Korea; Jeju National University, KOREA, REPUBLIC OF

## Abstract

Single nucleotide polymorphism (SNP) genotyping has revolutionized crop improvement by enabling high-resolution genomic analyses and accelerating breeding programs. In soybean (*Glycine max* (L.) Merr.), a globally important legume crop, existing genotyping data for 180,961 SNP markers from the Korean soybean core collection were generated using the outdated Williams 82 reference genome version 1 (Wm82.v1), which contains numerous assembly gaps and misassemblies that limit genomic resolution. While high-quality reference genomes including Wm82.v4 and Wm82.v6 (telomere-to-telomere assembly) are now available, the valuable existing SNP array data have not been integrated with these improved genomic resources. Here we show successful remapping of the 180K SNP array data to both Wm82.v4 and Wm82.v6 reference genomes through sequence-based alignment of flanking regions. We extracted flanking sequences from SNP marker positions in Wm82.v1 and mapped them to the newer reference versions based on sequence similarity, excluding markers with mapping failures, allele mismatches, low-identity alignments, or multiple mappings, which resulted in the successful mapping of 175,202 and 175,763 markers to Wm82.v4 and Wm82.v6, respectively. We also remapped genotype data from 927 soybean accessions (497 USDA-GRIN accessions and 430 Korean core collection accessions) to both reference versions. This updated SNP dataset provides the soybean research community with a comprehensive genomic resource that leverages both existing genotyping investments and state-of-the-art reference genome assemblies for enhanced crop improvement and genomic studies.

## Introduction

Soybean (*Glycine max* (L.) Merr.), a major crop worldwide, is an essential source of protein and oil for human nutrition, animal feed, and industrial applications. Advances in genomic technologies have substantially improved our understanding of soybean genetics, aiding in the identification of traits linked to yield, stress resistance, and quality improvement [1–2]. Among these technologies, single nucleotide polymorphism (SNP) arrays have enabled high-throughput genotyping and trait mapping in soybean breeding programs [3].

The 180K SNP array [4], developed using the Williams 82 (Wm82) genome version 1 (Wm82.v1) and version 2 (Wm82.v2) [5], is a pivotal resource for genetic studies in the Korean soybean core collection [6]. However, the Wm82.v1 and Wm82.v2 genomes, published in the early stages of soybean genomics, are characterized by incomplete assemblies and structural errors that limit the resolution and accuracy of downstream analyses. These challenges are compounded by the palaeopolyploid nature of the soybean genome, which includes extensive duplications and structural rearrangements from past whole-genome duplications. The use of short-read sequencing technologies further constrained the ability of the assembly to resolve repetitive and complex regions, leaving significant gaps and misassemblies.

The evolution of sequencing technologies, particularly the advent of long-read and high-fidelity sequencing, has dramatically improved the quality of genome assemblies. These advances have paved the way for telomere-to-telomere (T2T) genome assemblies, which provide unprecedented continuity and completeness. In recent years, T2T-level assemblies have been actively generated in a wide range of plant species, underscoring a growing trend in plant genomics to establish gap-free reference genomes that capture structural variation and haplotype diversity [7–8]. T2T genomes have enabled novel biological insights by resolving previously inaccessible genomic regions. For instance, in grapevine (*Vitis vinifera*), haplotype-resolved T2T assemblies revealed regulatory elements underlying anthocyanin accumulation, thereby clarifying the genetic basis of berry coloring [9]. Similarly, in rice (*Oryza sativa*), gap-free assemblies of multiple parental lines uncovered structural variations associated with heterosis, which had remained elusive in earlier draft genomes [10].

In soybean, successive releases of the Williams 82 reference genome illustrate this trajectory, with version 4 (Wm82.v4) marking a substantial improvement and version 6 (Wm82.v6) achieving a T2T level of completeness [11–12]. From previous research, genome upgrades enhanced assembly quality. The number of scaffolds was reduced from 3,363 in Wm82.v1 to 282 in Wm82.v4 and further to 20 in Wm82.v6, thereby achieving a chromosome-level assembly that fully corresponds to the 20 soybean chromosomes. Similarly, the contiguity of the assembly improved, with the scaffold N50 increasing from 419 kb in Wm82.v4 to 44.5 Mb in Wm82.v6 [12]. These improvements resolved many structural issues inherent to Wm82.v1, thus providing a more robust framework for genomic analysis. However, despite the availability of high-quality genome assemblies, many legacy SNP datasets remain anchored to outdated references. This disconnect hinders their integration with modern genomic tools.

Thus, this study aimed to reposition the 180K SNP array dataset, which was originally genotyped on older versions of the Wm82 genome, onto the latest versions 4 and 6. By performing this liftover process, we sought to improve the accuracy of SNP positions, resolve inconsistencies arising from genome version differences, and ensure compatibility with modern genomic analyses. Repositioning of these datasets is expected to enhance the reliability of marker-trait associations, optimize genomic selection strategies in breeding programs, and facilitate functional genomic studies by aligning genotypic data with the most up-to-date reference genomes.

## Materials and methods

### Data collection

Affymetrix Axiom® 180K SoyaSNP array data were collected from the previous study [4]. The genotype data of 4,234 soybean accessions, derived from the Affymetrix Axiom® 180K SoyaSNP array, were collected from the previous study [6]. The genotype data of 430 Korean soybean core collection and 497 plant introduction (PI) soybean collection were obtained from the dataset of 4,234 soybean accessions. Various versions of the Wm82 genome and genes were collected from SoyBase (www.soybase.org) [13]. Transposable element (TE) sequences [14] of soybean were also collected from SoyBase.

### 180K Axiom® SoyaSNP array liftover

To update the 180K SoyaSNP array data to Wm82.v4 and Wm82.v6, we employed the tools, parameters, and procedures outlined in this study (S1 Table). To liftover the 180K SoyaSNP array data to each Wm82 genome, we extracted the 50 bp flanked upstream and downstream sequences at the SNP position of Wm82.v1 using BEDTools getfasta [15]. BLAT [16] was employed to identify homologous regions surrounding the target SNPs. Flanking sequences from the Wm82.v1 assembly were aligned against other Wm82 assembly versions using the parameters -tileSize = 12, -minScore = 100, and -minIdentity = 98. The UCSC axtChain was used to chain the alignment blocks and identify the collinearity between genomes [17]. Of the 180K SNP data, 170K SNPs representing genetic variation within the Korean soybean core collection in VCF format were lifted using the Picard tool, LiftoverVcf (v2.22.8), and chain information (http://picard.sourceforge.net/). The remaining SNPs of the 180K data were lifted based on positional information from the chain file.

### Liftover validation

The flanking sequences of SNPs redundantly aligned to multiple locations between Wm82.v4 and Wm82.v6 were filtered. The lifted SNPs were manually validated using an in-house script, in which flanking sequences were first extracted from each genome assembly using SAMtools faidx [18]. Both forward and reverse strands were considered, and sequences were compared base by base, retaining only those SNP markers that exhibited 100% identity between the source and target assemblies. After the validation, flanking sequences of SNPs that show identity less than 100% between Wm82.v1 and the target Wm82 genome sequence were filtered. The density of the genes and lifted markers at Wm82.v6 were visualized using the RIdeogram package in R [19]. BEDTools complement was used to categorize lifted markers genic (within gene regions), near-genic (within 5 kbp upstream or downstream of a gene), or intergenic (all other regions) at Wm82.v4 and Wm82.v6. The R package circlize was used to visualize the distribution of unmapped markers within TE regions of Wm82.v1 and genomic low-identity regions between assemblies [20]. Gene synteny between Wm82.v4 and Wm82.v6 was identified using MCScan with JCVI toolkits [21]. The NUCmer package of MUMmer was employed to align the intergenic regions between Wm82.v4 and Wm82.v6 [22]. Syri was used to identify genomic rearrangements and collinear regions between genomes [23]. JBrowse2 was employed to visualize the microstructure of each genome [24]. RepeatMasker was used to identify the location of TEs [25]. NABIS 2, a high-performance computer from the Rural Development Administration of South Korea, was utilized for the computational analysis.

### Statistical analysis

Minor allele frequencies (MAFs) for each marker position in the soybean collection’s genotype data were calculated using an in-house script.

## Results

### Liftover of 180K Axiom® SoyaSNP array

**Fig 1:**
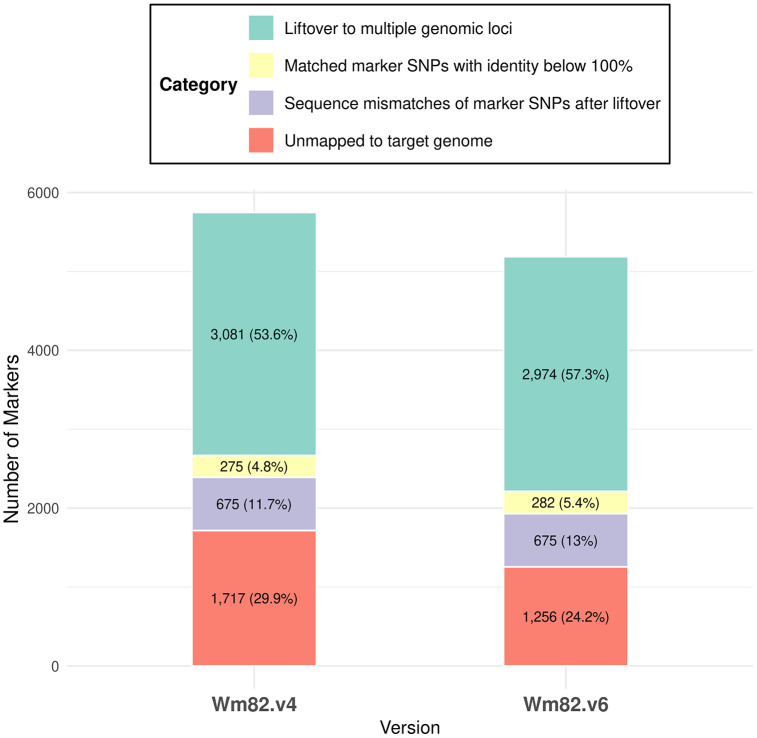
Overview of SNP markers with liftover failures.

Of the 180,950 SNP markers from the Affymetrix Axiom® 180K SoyaSNP array genotyped onto Wm82.v1, 5,748 and 5,187 markers failed to be lifted to Wm82.v4 and Wm82.v6, respectively (Fig 1). Of these unmapped markers, 3,081 in Wm82.v4 and 2,974 in Wm82.v6 failed because of mapping to multiple genomic loci, and 1,717 and 1,256 were completely unmapped to the target genome. By contrast, 176,152 and 176,720 markers were initially lifted to Wm82.v4 and Wm82.v6, respectively. After the initial liftover, an additional 675 markers were filtered from both Wm82.v4 and Wm82.v6 owing to SNP allele divergence. In addition, 275 and 282 markers that did not exhibit 100% identity with the Wm82.v1 genome were filtered at Wm82.v4 and Wm82.v6. The 275 markers that failed to be lifted to Wm82.v4 showed sequence identities ranging from 50% to 70%. Meanwhile, 280 markers among the 282 markers that failed to be lifted to Wm82.v6 showed sequence identities ranging from 50% to 70%, and the 2 remaining markers had sequence identities ranging from 70% to 90% (S2 Table). The total number of retained markers did not significantly differ from the results obtained with a 100% identity threshold. Ultimately, 175,202 and 175,763 markers were retained in Wm82.v4 and Wm82.v6, respectively (Table 1).

**Table 1:** Summary of the 180K SNP array data liftover.

Chromosome	Total SNPs (Wm82.v1)^*ab*^		Lifted SNPs											
			Wm82.v2^*ab*^				Wm82.v4				Wm82.v6			
			Successfully lifted		Failed to lift		Successfully lifted		Failed to lift		Successfully lifted		Failed to lift	
Chr01	8,935	4.94%	8,379	4.63%	556	0.31%	8,797	4.86%	138	0.08%	8,833	4.88%	102	0.06%
Chr02	10,224	5.65%	9,514	5.26%	710	0.39%	9,857	5.45%	367	0.20%	9,885	5.46%	339	0.19%
Chr03	8,417	4.65%	7,673	4.24%	744	0.41%	7,737	4.28%	680	0.38%	7,772	4.29%	645	0.36%
Chr04	8,638	4.77%	8,399	4.64%	239	0.13%	8,567	4.73%	71	0.04%	8,597	4.75%	41	0.02%
Chr05	8,024	4.43%	7,577	4.19%	447	0.25%	7,653	4.23%	371	0.21%	7,727	4.27%	297	0.16%
Chr06	9,906	5.47%	9,392	5.19%	514	0.28%	9,607	5.31%	299	0.17%	9,693	5.36%	213	0.12%
Chr07	8,588	4.75%	8,126	4.49%	462	0.26%	8,209	4.54%	379	0.21%	8,234	4.55%	354	0.20%
Chr08	10,996	6.08%	10,475	5.79%	521	0.29%	10,712	5.92%	284	0.16%	10,777	5.96%	219	0.12%
Chr09	8,996	4.97%	8,621	4.76%	375	0.21%	8,912	4.92%	84	0.05%	8,962	4.95%	34	0.02%
Chr10	9,309	5.14%	8,820	4.87%	489	0.27%	9,099	5.03%	210	0.12%	9,153	5.06%	156	0.09%
Chr11	8,412	4.65%	7,481	4.13%	931	0.51%	8,172	4.52%	240	0.13%	8,222	4.54%	190	0.11%
Chr12	7,708	4.26%	7,222	3.99%	486	0.27%	7,495	4.14%	213	0.12%	7,520	4.16%	188	0.10%
Chr13	10,885	6.02%	10,338	5.71%	547	0.30%	10,595	5.85%	290	0.16%	10,687	5.91%	198	0.11%
Chr14	7,817	4.32%	7,195	3.98%	622	0.34%	7,560	4.18%	257	0.14%	7,604	4.20%	213	0.12%
Chr15	10,136	5.60%	9,423	5.21%	713	0.39%	9,835	5.43%	301	0.17%	9,864	5.45%	272	0.15%
Chr16	7,590	4.19%	6,980	3.86%	610	0.34%	7,348	4.06%	242	0.13%	7,367	4.07%	223	0.12%
Chr17	8,906	4.92%	8,347	4.61%	559	0.31%	8,663	4.79%	243	0.13%	8,682	4.80%	224	0.12%
Chr18	9,957	5.50%	9,103	5.03%	854	0.47%	9,518	5.26%	439	0.24%	9,576	5.29%	381	0.21%
Chr19	8,719	4.82%	8,166	4.51%	553	0.31%	8,480	4.69%	239	0.13%	8,503	4.70%	216	0.12%
Chr20	8,212	4.54%	7,797	4.31%	415	0.23%	8,072	4.46%	140	0.08%	8,105	4.48%	107	0.06%
Scaffolds	575	0.32%	1,185	0.65%	–	–	314	0.17%	261	0.14%	0	0.00%	0	0.00%
**Total**	180,950	–	170,213	94.07%	10,737	5.93%	175,202	96.82%	5,748	3.18%	175,763	97.13%	5,187	2.87%

^a^A data previously reported [4].

^b^Chloroplast markers were excluded.

**Fig 2:**
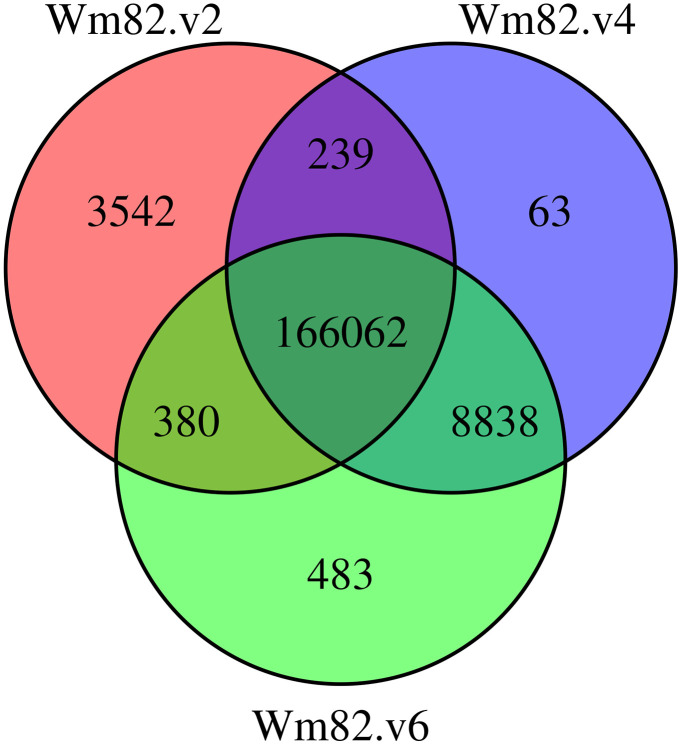
Venn diagram of the lifted SNP marker across the various versions of Wm82 genomes.

The liftover rates of the markers in the Wm82.v4 and Wm82.v6 genomes were 96.82% and 97.13%, respectively. Among the lifted markers, 162,020 and 162,758 were lifted as forward strands, whereas 13,182 and 13,005 were lifted as reverse strands in Wm82.v4 and Wm82.v6, respectively (S3 Table). Among all the lifted markers, 166,062 were successfully mapped to Wm82.v2, Wm82.v4, and Wm82.v6 (Fig 2). Moreover, 9,457 markers were lifted to at least two different Wm82 versions. Specifically, 8,838 markers were lifted to both Wm82.v4 and Wm82.v6, 380 to Wm82.v2 and Wm82.v6, and 239 to Wm82.v2 and Wm82.v4. Additionally, 4,088 markers were uniquely lifted to a single Wm82 version, including 3,542 markers to Wm82.v2, 63 markers to Wm82.v4, and 483 markers to Wm82.v6. Furthermore, 1,354 markers could not be lifted to any of the Wm82.v2, Wm82.v4, and Wm82.v6 versions.

For chromosome-level comparison, we evaluated the number of markers retained on the same chromosome from Wm82.v1 to the target genome (S4 Table). Of the 8,935 markers genotyped on chromosome 1 of Wm82.v1 in a previous study, 97.88% and 98.27% of the markers were lifted to chromosome 1 in Wm82.v4 and Wm82.v6, which exhibited the highest success rates among the chromosomes. Of these, 8,746 and 8,780 markers were originally from chromosome 1, whereas 51 and 53 were lifted from the Wm82.v1 scaffolds. By contrast, chromosome 3 showed the lowest liftover rates of 91.85% and 92.17% for Wm82.v1 markers lifted to Wm82.v4 and Wm82.v6, respectively. The most significant difference in liftover rates between Wm82.v4 and Wm82.v6 was observed on chromosome 6, with rates of 96.64% and 97.70%, respectively, reflecting a 1.06% difference. Finally, we identified markers that were originally genotyped on scaffolds in Wm82.v1 but were lifted over to chromosomes in Wm82.v4 and Wm82.v6 (S5 Table). Of 575 markers of the Wm82.v1 scaffolds, 51 markers remained at the Wm82.v4 scaffolds, while 350 markers were lifted to the chromosome. However, in Wm82.v6, a T2T-level genome without any scaffold, 456 markers were lifted from the scaffolds to the chromosome. Among the originally genotyped markers on scaffolds in Wm82.v1, a total of 174 and 119 markers failed to be lifted to Wm82.v4 and Wm82.v6, respectively.

### Application of SNP liftover to the soybean collections

Among the 175,202 and 175,763 SNP markers that were lifted to Wm82.v4 and Wm82.v6, 166,301 and 166,442, respectively, were present among the 170,223 markers genotyped in the soybean collection from Wm82.v1. As a result, without duplicates, 166,681 markers were lifted to the markers of the soybean collection. In total, 166,062 markers were lifted to both Wm82.v4 and Wm82.v6, whereas 239 and 380 markers were lifted separately.

For comparison, the lifted markers were classified based on allele frequency. We first examined the allele frequency in the 430 Korean soybean core collection (Table 2). A total of 30,487 and 30,509 markers from Wm82.v4 and Wm82.v6, respectively, which were lifted to the 430 Korean soybean core collection, exhibited monomorphic alleles. Additionally, 32,511 and 32,554 markers had MAFs below 1%; 23,044 and 23,049 between 1% and 5%; 17,144 and 17,170 within the 5%–10% range; and 24,279 and 24,309 between 10% and 20%. In total, 14,179 and 14,734 markers with MAFs between 20% and 30%; 12,792 and 12,796 within the 30%–40%; 11,325 and 11,321 greater than 40% were lifted to Wm82.v4 and Wm82.v6, respectively. The markers with MAFs lower than 1% (approximately 19.55% of the total markers) were predominantly lifted at both Wm82.v4 and Wm82.v6. Except for markers with MAFs greater than 40%, the number of markers lifted to Wm82.v6 was slightly higher than that lifted to Wm82.v4, with the largest difference observed for rare alleles.

**Table 2:** Number of lifted markers to the 430 Korean soybean core collection categorized by MAF.

MAF	Number of SNPs			
	Wm82.v4		Wm82.v6	
Monomorphic	30,487	18.33%	30,509	18.33%
<0.01	32,511	19.55%	32,554	19.56%
0.01 ~ 0.05	23,044	13.86%	23,049	13.85%
0.05 ~ 0.10	17,144	10.31%	17,170	10.32%
0.10 ~ 0.20	24,279	14.60%	24,309	14.61%
0.20 ~ 0.30	14,719	8.85%	14,734	8.85%
0.30 ~ 0.40	12,792	7.69%	12,796	7.69%
0.40>	11,325	6.81%	11,321	6.80%
**Total**	166,301	–	166,442	–

Next, we analyzed allele frequencies in the 497 PI soybean collection (Table 3). Among the total lifted markers, 11,636 and 11,642 mapped to Wm82.v4 and Wm82.v6, respectively, corresponding to 7.00% of the dataset, were classified as monomorphic alleles. Furthermore, 19,771 and 19,805 markers were classified as a rare allele, which exhibited MAFs lower than 1%, 36,246 and 36,256 between 1%–5%, 24,003 and 24,037 within 5%–10%, and 31,202 and 31,236 within 10%–20%. The number of markers with MAFs between 20% and 30% totaled 17,361 in Wm82.v4 and 17,377 in Wm82.v6; 13,516 and 13,530 within 30%–40%; and 12,566 and 12,559 greater than 40%, respectively. Unlike the Korean soybean core collection, in which rare alleles predominated, the PI soybean collection was dominated by markers with MAFs between 1% and 5%, accounting for ~21.80% of the total. In addition, the greatest difference between the two collections was observed in monomorphic alleles: approximately 7% of alleles in the PI soybean collection were monomorphic, whereas this proportion was about 18% in the Korean soybean core collection. Among the markers lifted to the 430 Korean soybean core collection and the 497 PI soybean collection, we also observed MAFs of markers that were uniquely lifted over to Wm82.v4 and Wm82.v6, respectively. The MAFs of rare alleles were higher in markers lifted over to Wm82.v6 in both collections (S6 and S7 Tables). Among the 380 uniquely lifted markers in Wm82.v6, 90 and 58 markers from the 430 Korean soybean core collection and 497 PI soybean core were classified as rare alleles, of which 47 and 24 were identified among the 239 uniquely lifted markers in Wm82.v4. Furthermore, the higher proportion of monomorphic alleles observed in the Korean soybean core collection compared with the PI collection was also evident among uniquely lifted markers. Among the uniquely lifted markers, approximately 16% were monomorphic in the Korean soybean core collection, compared with about 10% in the PI soybean collection.

**Table 3:** Number of lifted markers to the 497 PI soybean collection categorized by MAF.

MAF	Number of SNPs			
	Wm82.v4		Wm82.v6	
Monomorphic	11,636	7.00%	11,642	6.99%
<0.01	19,771	11.89%	19,805	11.90%
0.01 ~ 0.05	36,246	21.80%	36,256	21.78%
0.05 ~ 0.10	24,003	14.43%	24,037	14.44%
0.10 ~ 0.20	31,202	18.76%	31,236	18.77%
0.20 ~ 0.30	17,361	10.44%	17,377	10.44%
0.30 ~ 0.40	13,516	8.13%	13,530	8.13%
0.40>	12,566	7.56%	12,559	7.55%
**Total**	166,301	–	166,442	–

Finally, we analyzed the differences in the distribution of rare alleles observed between the PI soybean collection and the Korean soybean core collection. We examined how rare alleles identified in each collection were represented in the other collection in terms of allele frequency (S8 and S9 Tables). Of the 19,829 and 32,601 rare alleles in the PI and Korean soybean collections, 9,651 markers were classified as rare in both collections. In contrast, 10,178 markers from the PI collection and 22,950 markers from the Korean collection were classified as non-rare in the other collection. Notably, the most pronounced difference was observed in monomorphic alleles: 6.10% of markers from the Korean collection were monomorphic in the PI collection, whereas 46.27% of markers from the PI collection were monomorphic in the Korean soybean core collection.

### Classification of lifted SNP array based on genomic features

**Fig 3:**
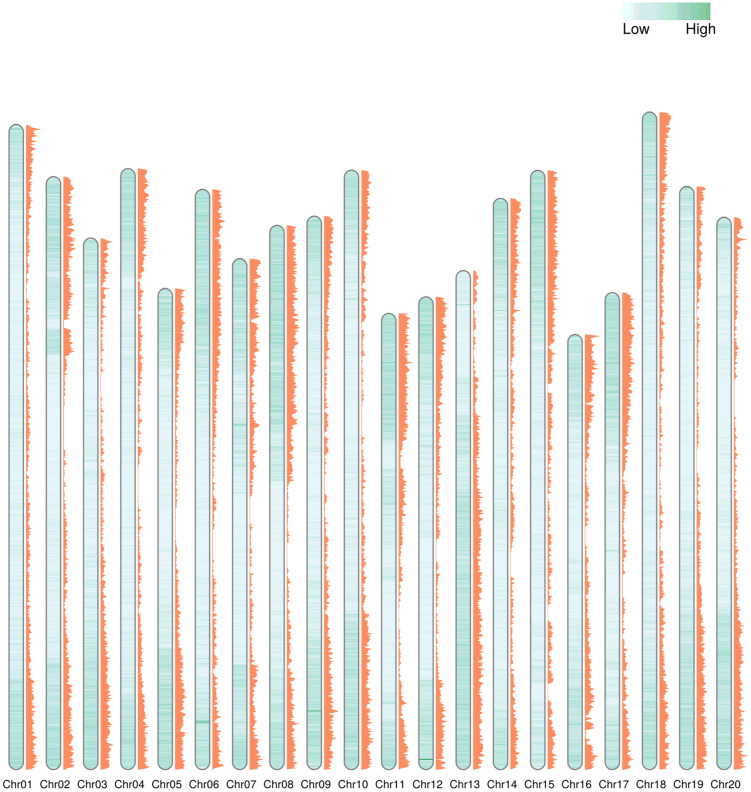
Comprehensive visualization of the gene distribution and lifted SNP marker landscape in Wm82.v6. The inner green heatmap represents gene density along the chromosomes, while the adjacent orange histogram illustrates the density of lifted SNP markers.

Subsequently, we analyzed the positional characteristics of the SNP markers following liftover, focusing on their physical locations across chromosomes. The 175,202 and 175,763 markers lifted to Wm82.v4 and Wm82.v6, respectively, were predominantly located in the gene-rich chromosome arm regions (Fig 3).

**Fig 4:**
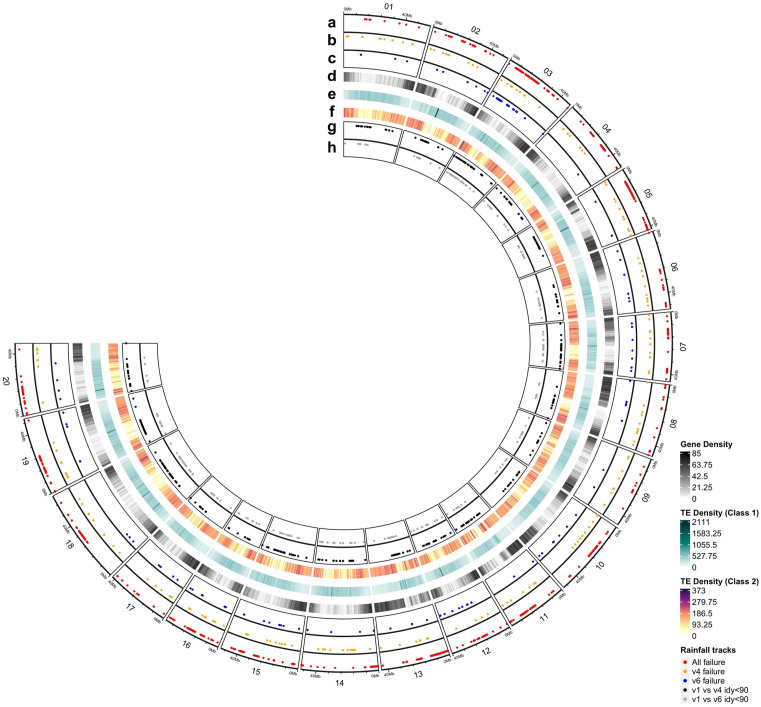
Visualization of SNP array markers with liftover failures. The plot is based on the Wm82.v1 genome, with scaffold marker information excluded. **(a)** Markers with liftover failure in all genome versions. **(b)** Markers with liftover failure only in Wm82.v4. **(c)** Markers with liftover failure only in Wm82.v6. **(d)** Gene density heatmap. **(e)** Density heatmap of Class I transposons in Wm82.v1. **(f)** Density heatmap of Class II transposons in Wm82.v1. **(g)** Genomic regions with <90% identity between Wm82.v1 and Wm82.v4. **(h)** Genomic regions with <90% identity between Wm82.v1 and Wm82.v6.

For a more detailed classification, the lifted markers were categorized as genic (within gene regions), near-genic (within 5 kbp upstream or downstream of a gene), or intergenic (all other regions) (Table 4). Markers lifted to Wm82.v4 and Wm82.v6 were predominantly located in genic regions. A total of 110,926 and 111,261 markers from Wm82.v4 and Wm82.v6, respectively, with approximately 60% of the total lifted markers, were located in genic regions. The next most common categories were intergenic and near-genic regions, with markers lifted to these regions in descending order of frequency. Among the three categories of lifted markers, the largest difference in liftover rates between Wm82.v4 and Wm82.v6 was observed in intergenic regions. In total, 37,492 and 39,066 markers were lifted to the intergenic regions of Wm82.v4 and Wm82.v6, representing 20.72% and 21.59%, respectively. Next, we identified the 5,748 and 5,187 markers that failed to be lifted to Wm82.v4 and Wm82.v6, respectively, which were already categorized as genic, intergenic, upstream, and downstream using Wm82.v1 from previous studies. Among the markers that failed to be lifted to both the Wm82.v4 and Wm82.v6 genome assemblies, the majority were predominantly located within the genic regions of Wm82.v1. A total of 3,548 and 3,329 markers were located in genic regions; 1,419 and 1,193 in intergenic regions; 263 and 219 in upstream regions; and 518 and 454 in downstream regions of Wm82.v1. In addition, we further classified the unmapped SNP markers into three categories: markers that failed to lift across all Wm82 versions, markers that failed only in Wm82.v4, and markers that failed only in Wm82.v6 (Table 5). A total of 1,354, 380, and 239 markers, respectively, were assigned to these categories. Across all three categories, intergenic regions exhibited the highest rates of liftover failure, with 756, 183, and 135 markers failing in Wm82.v1 intergenic regions. To investigate the causes of these failures, we examined the presence of TEs and low-identity regions between assemblies (Table 6 and Fig 4). Although nearly half of the markers were unrelated to TEs or low-identity between genomic regions, 48.67%, 37.63%, and 35.15% of the markers in the three categories, respectively, were found to be nested within retrotransposons.

**Table 4:** Distribution of the 180K SNP array markers at the various Wm82 genome versions.

SNP Type	Region	Number of SNPs							
		Wm82.v1^*ab*^		Wm82.v2^*ab*^		Wm82.v4		Wm82.v6	
Lifted	Genic^*c*^	114,735	63.40%	111,154	61.42%	110,926	61.30%	111,261	61.48%
	Intergenic^*e*^	43,263	23.91%	32,751	18.10%	37,492	20.72%	39,066	21.59%
	Near-genic^*d*^	22,952	12.68%	26,308	14.54%	26,784	14.80%	25,436	14.06%
	**Total**	180,950	100%	170,213	94.07%	175,202	96.82%	175,763	97.13%
Non- lifted	Upstream^*d*^	–	–	746	0.41%	263	0.15%	211	0.12%
	Genic^*c*^	–	–	4,559	2.52%	3,548	1.96%	3,329	1.84%
	Intergenic^*e*^	–	–	4,190	2.32%	1,419	0.78%	1,193	0.66%
	Downstream^*d*^	–	–	1,242	0.69%	518	0.29%	454	0.25%
	**Total**	–	–	10,737	5.93%	5,748	3.18%	5,187	2.87%

^a^A data previously reported [4].

^b^Chloroplast markers were excluded.

^c^Located within gene regions.

^d^Outside of gene regions but within 5 kbp upstream or downstream.

^e^Non-genic, non-near-genic regions.

**Table 5:** Distribution of the 180K SNP array markers with liftover failure.

Region (Wm82.v1)	Number of SNPs					
	Failed to every genome^*ab*^		Failed only Wn82.v4		Failed only Wn82.v6	
Upstream^*d*^	62	4.58%	27	7.11%	7	2.93%
Genic^*c*^	437	32.27%	132	34.74%	73	30.54%
Intergenic^*e*^	756	55.83%	183	48.16%	135	56.49%
Downstream^*d*^	99	7.31%	38	10.00%	24	10.04%
**Total**	1,354	–	380	–	239	–

^a^A data of Wm82.v2 was previously reported [4].

^b^Chloroplast markers were excluded.

^c^Located within gene regions.

^d^Outside of gene regions but within 5 kbp upstream or downstream.

^e^Non-genic, non-near-genic regions.

**Table 6:** Characteristics of SNP array markers with liftover failure in Wm82 genome versions.

Region (Wm82.v1)	Number of SNPs					
	Failed to every genome^*ab*^		Failed only Wn82.v4		Failed only Wn82.v6	
Class I (Retrotransposon)	659	48.67%	143	37.63%	84	35.15%
Class II (DNA transposon)	102	7.53%	30	7.89%	9	3.77%
Identity below 95% to Wm82.v4	6	0.44%	0	0.00%	0	0.00%
Identity below 95% to Wm82.v6	7	0.52%	2	0.53%	0	0.00%
Others	580	42.84%	205	53.95%	146	61.09%
**Total**	1,354		380		239	

^a^A data of Wm82.v2 was previously reported [4].

^b^Chloroplast markers were excluded.

### Marker-lifted region comparison between the genomes

**Fig 5:**
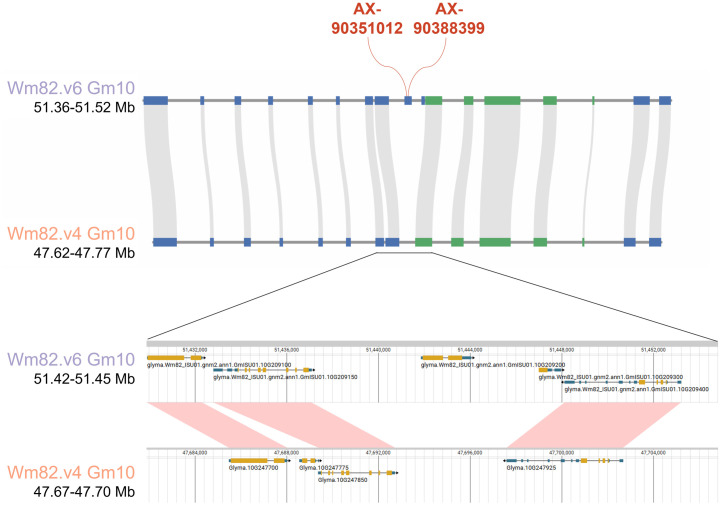
Visualization of genic regions for the liftover analysis comparing Wm82.v4 and Wm82.v6. Each block on the track represents the position of the gene. Gray and pink lines between tracks indicate syntenic regions, while the red line on the Wm82.v6 track marks the position of the genic marker.

To compare Wm82.v4 and Wm82.v6, we visualized the genic SNP markers and their surrounding regions using their positions (Fig 5). Among the 863 markers that failed to be lifted to Wm82.v4, we focused on two markers: AX-90351012 and AX-90388399. These markers were previously classified as genic markers in Wm82.v1, and their associated genes were located in unplaced contigs in both Wm82.v1 and Wm82.v2. However, in Wm82.v6, these unplaced contigs and their corresponding markers were assigned to chromosome 10. Specifically, AX-90351012 and AX-90388399 were located within the gene *GmISU01.10G209200* in Wm82.v6. Synteny analysis revealed that the genes flanking *GmISU01.10G209200* were conserved between Wm82.v4 and Wm82.v6, although *GmISU01.10G209200* and *GmISU01.10G209300*, the genes nearest to *GmISU01.10G209200*, were present only in Wm82.v6.

**Fig 6:**
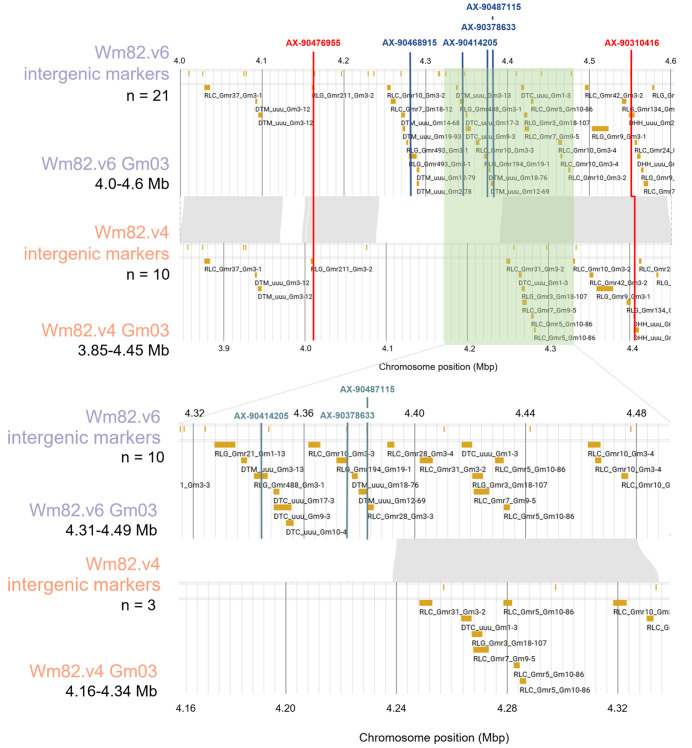
Visualization of intergenic marker regions for the liftover analysis comparing Wm82.v4 and Wm82.v6. The semi-transparent green blocks indicate zoomed-in regions. Gray regions between tracks indicate syntenic regions. The red lines on the track indicate TE nested intergenic markers specific to Wm82.v6, while the blue lines represent those present in both Wm82.v4 and Wm82.v6.

To compare the intergenic regions between Wm82.v4 and Wm82.v6, we focused on the 3.85–4.45 and 4.00–4.60 Mbp regions on chromosome 3 from both genomes (Fig 6). In this region, 23 TEs longer than 2 kbp and 10 intergenic markers were identified in Wm82.v4, whereas 43 TEs longer than 2 kbp and 21 intergenic markers were identified in Wm82.v6. Among the 21 intergenic markers in Wm82.v6, 10 were located in regions homologous to Wm82.v4, whereas 11 were located in unique regions. Additionally, among the 21 intergenic markers of Wm82.v6 mapped to this region, six markers were nested within TEs. These marker-associated TEs were identified as belonging to the Gypsy retrotransposon superfamilies, as well as the Mutator and Helitron DNA transposon superfamilies. Of the six TE-nested markers, two were common between Wm82.v4 and Wm82.v6 and were located in homologous regions. The four remaining markers were uniquely mapped to Wm82.v6, which resides in unique regions.

## Discussion

Williams 82, the soybean reference genome, was first assembled in 2010 and has since played a crucial role in advancing genomic studies within the soybean research community [5]. Early genotyping efforts, such as the 180K SoyaSNP project, relied on this genome for marker-based analyses [4]. However, the reference genome is continuously updated, and many of these markers remain anchored to early genome versions. This limits their utility in genomic studies. As genome assemblies have improved, particularly with the advent of T2T-level reference genomes, reassigning these markers to more complete assemblies has become increasingly important. In this study, we performed marker liftovers to Wm82.v4 and Wm82.v6, updated the marker positions to high-quality reference genomes, and emphasized the necessity of using the latest genome versions with the highest structural accuracy.

The liftover from Wm82.v1 to Wm82.v4 and Wm82.v6 revealed slightly higher success rates for Wm82.v6 on each chromosome. While most markers were retained on the same chromosomes across both versions, Wm82.v6 exhibited better liftover success, particularly for certain chromosomes. This improvement emphasizes the high accuracy and utility of Wm82.v6 for genomic analyses. However, a small proportion of the markers that failed to be lifted to any version, possibly reflecting structural variations in the genome. These results underscore the need for updated reference genomes such as Wm82.v6 to ensure more precise and reliable genomic studies in soybean research.

The SNP markers lifted from the soybean collection to Wm82.v4 and Wm82.v6 further illustrate the value of updated reference genomes in capturing genetic diversity. Wm82.v6, in particular, showed a slightly higher number of lifted markers across all MAF ranges, and this trend was observed in both the Korean and PI collections. While the Korean collection contained a higher number of rare alleles, it also exhibited a greater proportion of monomorphic alleles compared with the PI soybean collection. Consistently, approximately 64% of rare alleles in the Korean collection were classified as non-rare polymorphic alleles in the PI soybean collection, whereas only 5% of rare alleles in the PI soybean collection were classified as non-rare polymorphic in the Korean collection. These patterns support the higher genetic diversity in the PI soybean collection. Considering the characteristics of each collection, this difference in genetic diversity may be attributable to their origins. The Korean soybean core collection primarily comprises accessions from Korea, which may have undergone population-specific selection pressures, whereas the PI soybean collection represents a broader geographic range. Furthermore, the allele distribution across the Williams 82 versions also highlights the importance of using high-quality genome assemblies, such as Wm82.v6, in detecting rare alleles and improving our understanding of genetic variation. Overall, the improved retention of rare alleles in Wm82.v6 underscores the importance of using the most complete reference genome for marker-based analyses and population genetic studies.

The genomic distribution of the lifted SNP markers also highlights the broader impact of reference genome updates on marker positioning. Generally, Wm82.v6 retained a higher proportion of markers in both genic and intergenic regions than Wm82.v4, and fewer markers that failed to lift overall, suggesting improved assembly accuracy. In both Wm82.v4 and Wm82.v6, the majority (approximately 60%) of the lifted markers were located within genic regions, reflecting the genic-rich design of the original array, in which 63% of the markers resided in genic regions [4]. Although gene predictions differ across all Wm82 versions, the array still shows a similar genic preference; in some cases, markers are anchored within newly predicted genes. Despite the importance of detecting variants in intergenic and near-genic regions (e.g., promoters and enhancers) for capturing regulatory effects [26–28], the uneven distribution of the SNP array limits coverage in these regions, potentially hindering the detection of regulatory variants associated with traits [29]. Moreover, markers that failed to lift across all Wm82 versions—or only in specific versions—were predominantly located in intergenic regions of Wm82.v1, particularly within retrotransposons. The enrichment of failed markers in these repetitive regions underscores the intrinsic difficulty of achieving accurate SNP coordinate transfer across genome versions. In addition, the gene-rich design of the SNP array itself limits uniform genome-wide genotype resolution, reflecting both the structural challenges of the genome and the intrinsic biases of the marker set. These factors should be considered when interpreting liftover results and planning genotyping strategies.

Building on these observations, the comparison between Wm82.v4 and Wm82.v6 revealed notable improvements in marker liftover and genomic distribution. Markers such as AX-90351012 and AX-90388399, previously located on unplaced contigs in Wm82.v1, were successfully assigned to chromosome 10 in Wm82.v6. Synteny analysis showed that the genes flanking *GmISU01.10G209200* were conserved between the two versions, whereas the additional genes in Wm82.v6 highlighted its greater completeness than Wm82.v4. Furthermore, when comparing intergenic regions on chromosome 3, Wm82.v6 showed a higher number of TEs and intergenic markers than Wm82.v4, many of which were uniquely lifted or nested within TEs. Our results further underscore the superior genomic resolution and annotation accuracy of Wm82.v6, reinforcing the importance of using the latest reference genomes for more precise genetic analyses. By repositioning the markers based on the high-precision Wm82.v6 genome, we anticipate their usefulness in further genome-wide association studies and marker-assisted selection breeding efforts. Owing to the high accuracy inherent in T2T-level genomes as shown in sorghum and spinach [30–31], which anchored the unplaced contigs and curated misassemblies, SNP liftover onto these assemblies can be expected to correct SNPs that were previously mis-mapped or positioned on scaffolds. These SNPs based on high-quality genome can serve as valuable markers in genomic selection and genome-wide association studies, potentially improving prediction accuracy and trait association reliability [32]. Our updates based on the T2T-level genome are expected to facilitate the identification of previously ambiguous trait-associated genes by enabling direct correlation between newly predicted genes in the complete Wm82.v6 sequence and the lifted SNP array markers.

## Supporting information

S1 Table Summary of tools and parameters used in this study. (DOCX)

S2 Table Distribution of flanked marker sequence with identity below 100%. (DOCX)

S3 Table Orientation of lifted markers relative to Wm82.v1. (DOCX)

S4 Table Chromosome-level assessment of the 180K SNP array data liftover efficiency relative to Wm82.v1. (DOCX)

S5 Table Liftover status of predicted markers from scaffolds in Wm82.v1 to chromosomes across different Wm82 genome versions. (DOCX)

S6 Table Categorization of uniquely lifted markers to 430 Korean soybean core collection using MAF. (DOCX)

S7 Table Categorization of uniquely lifted markers to 497 PI soybean collection using MAF. (DOCX)

S8 Table Categorization of rare alleles from 430 Korean soybean core collection in 497 PI soybean collection. (DOCX)

S9 Table Categorization of rare alleles from 497 PI soybean collection in 430 Korean soybean core collection. (DOCX)
