# Supplementary material for: Updated resource of 180K soybean SNP genotyping array based on the T2T reference genome
Source: PLoS One. 2025 Dec 5;20(12):e0335227. doi: 10.1371/journal.pone.0335227 (PMC12680204; doi:10.1371/journal.pone.0335227)
Supplement: S1 Table — (DOCX) [file pone.0335227.s001.docx]

**S1 Table.**

| **Used Tools** | **Version** | **Key Parameters** | **Objective** |
| --- | --- | --- | --- |
| bedtools | v2.30.0 | getfasta | Extract FASTA  from bed file |
|  |  | complement, subtract, intersect | Categorize genomic regions |
| BLAT | v. 39x1 | tileSize=12  -minScore=100  -minIdentity=98 | Identify homologous  regions between genomes |
| axtChain | - | -linearGap=medium | chain the alignment blocks  and identify the collinearity |
| LiftoverVcf | v2.22.8 | Default parameters were used | Lift VCF file based on  chain information |
| SAMtools | v1.14 | faidx | Extract flanked SNP  sequence from the genome |
| JCVI | v1.0.10+4.ga37f702c | Default parameters were used | Identify the synteny |
| NUCmer | v4.0.0rc1 | --maxmatch -c 100 -b 500 -l 50 | Align the genome sequences |
| Syri | v1.7.0 | Default parameters were used | Identify genomic rearrangements  and collinear regions |
| RepeatMasker | v4.1.7-p1 | -xsmall -gff | Identify the location of  repeat sequences in genome |
