# Supplementary material for: Updated resource of 180K soybean SNP genotyping array based on the T2T reference genome
Source: PLoS One. 2025 Dec 5;20(12):e0335227. doi: 10.1371/journal.pone.0335227 (PMC12680204; doi:10.1371/journal.pone.0335227)
Supplement: S2 Table — (DOCX) [file pone.0335227.s002.docx]

**S2 Table.**

| **Identity Range (%)** | **Wm82.v4** | **Wm82.v6** |
| --- | --- | --- |
| 70–90 | 0 | 2 |
| 50–70 | 275 | 280 |
| **Total** | 275 | 282 |
