# Supplementary material for: Updated resource of 180K soybean SNP genotyping array based on the T2T reference genome
Source: PLoS One. 2025 Dec 5;20(12):e0335227. doi: 10.1371/journal.pone.0335227 (PMC12680204; doi:10.1371/journal.pone.0335227)
Supplement: S3 Table — (DOCX) [file pone.0335227.s003.docx]

**S3 Table.**

| **Strand** | **Number of SNPs** | | |
| --- | --- | --- | --- |
|  | **Wm82.v2*^ab^*** | **Wm82.v4** | **Wm82.v6** |
| Forward | 159,641 | 162,020 | 162,758 |
| Reverse | 10,572 | 13,182 | 13,005 |
| **Total** | 170,213 | 175,202 | 175,763 |

*^a^*A data previously reported [4].

*^b^*Chloroplast markers were excluded.
