# Supplementary material for: Updated resource of 180K soybean SNP genotyping array based on the T2T reference genome
Source: PLoS One. 2025 Dec 5;20(12):e0335227. doi: 10.1371/journal.pone.0335227 (PMC12680204; doi:10.1371/journal.pone.0335227)
Supplement: S5 Table — (DOCX) [file pone.0335227.s005.docx]

**S5 Table.**

| **Version** | **Wm82.v1*^a^****^b^* | **Wm82.v2*^a^****^b^* | | **Wm82.v4** | | **Wm82.v6** | |
| --- | --- | --- | --- | --- | --- | --- | --- |
| **Scaffold** | 575 | 142 | 24.70% | 51 | 8.87% | 0 | - |
| **Chromosome** | - | 313 | 54.43% | 350 | 60.87% | 456 | 79.30% |
| **Missing** | - | 120 | 20.87% | 174 | 30.26% | 119 | 20.70% |

*^a^*A data previously reported [4].

*^b^*Chloroplast markers were excluded.
