# Supplementary material for: Updated resource of 180K soybean SNP genotyping array based on the T2T reference genome
Source: PLoS One. 2025 Dec 5;20(12):e0335227. doi: 10.1371/journal.pone.0335227 (PMC12680204; doi:10.1371/journal.pone.0335227)
Supplement: S6 Table — (DOCX) [file pone.0335227.s006.docx]

**S6 Table.**

| **SNP Type** | **Minor Allele**  **Frequency** | **Number of SNPs** | | | |
| --- | --- | --- | --- | --- | --- |
|  |  | **Wm82.v4** | | **Wm82.v6** | |
| Lifted  to 430 Korean Soybean Core Collection | Monomorphic | 39 | 16.32% | 61 | 16.05% |
|  | <0.01 | 47 | 19.67% | 90 | 23.68% |
|  | 0.01~0.05 | 41 | 17.15% | 46 | 12.11% |
|  | 0.05~0.10 | 23 | 9.62% | 49 | 12.89% |
|  | 0.10~0.20 | 28 | 11.72% | 58 | 15.26% |
|  | 0.20~0.30 | 19 | 7.95% | 34 | 8.95% |
|  | 0.30~0.40 | 21 | 8.79% | 25 | 6.58% |
|  | 0.40> | 21 | 8.79% | 17 | 4.47% |
|  | **Total** | 239 |  | 380 |  |
