# Supplementary material for: Updated resource of 180K soybean SNP genotyping array based on the T2T reference genome
Source: PLoS One. 2025 Dec 5;20(12):e0335227. doi: 10.1371/journal.pone.0335227 (PMC12680204; doi:10.1371/journal.pone.0335227)
Supplement: S7 Table — (DOCX) [file pone.0335227.s007.docx]

**S7 Table.**

| **SNP Type** | **Minor Allele**  **Frequency** | **Number of SNPs** | | | |
| --- | --- | --- | --- | --- | --- |
|  |  | **Wm82.v4** | | **Wm82.v6** | |
| Lifted  to 497 PI Soybean Collection | Monomorphic | 28 | 11.72% | 34 | 8.95% |
|  | <0.01 | 24 | 10.04% | 58 | 15.26% |
|  | 0.01~0.05 | 55 | 23.01% | 65 | 17.11% |
|  | 0.05~0.10 | 27 | 11.30% | 61 | 16.05% |
|  | 0.10~0.20 | 42 | 17.57% | 76 | 20.00% |
|  | 0.20~0.30 | 20 | 8.37% | 36 | 9.47% |
|  | 0.30~0.40 | 17 | 7.11% | 31 | 8.16% |
|  | 0.40> | 26 | 10.88% | 19 | 5.00% |
|  | **Total** | 239 |  | 380 |  |
