# Supplementary material for: Updated resource of 180K soybean SNP genotyping array based on the T2T reference genome
Source: PLoS One. 2025 Dec 5;20(12):e0335227. doi: 10.1371/journal.pone.0335227 (PMC12680204; doi:10.1371/journal.pone.0335227)
Supplement: S8 Table — (DOCX) [file pone.0335227.s008.docx]

**S8 Table.**

| **SNP Type** | **Minor Allele**  **Frequency** | **Number of SNPs in PI soybean collection** | |
| --- | --- | --- | --- |
| 32,601 rare alleles in 430 Korean Soybean Core Collection | Monomorphic | 1,988 | 6.10% |
|  | <0.01 | 9,651 | 29.60% |
|  | 0.01~0.05 | 14,300 | 43.86% |
|  | 0.05~0.10 | 5,026 | 15.42% |
|  | 0.10~0.20 | 1,634 | 5.01% |
|  | 0.20~0.30 | 0 | 0.00% |
|  | 0.30~0.40 | 0 | 0.00% |
|  | 0.40> | 2 | 0.01% |
|  | **Total** | 32,601 |  |
