# Supplementary material for: Updated resource of 180K soybean SNP genotyping array based on the T2T reference genome
Source: PLoS One. 2025 Dec 5;20(12):e0335227. doi: 10.1371/journal.pone.0335227 (PMC12680204; doi:10.1371/journal.pone.0335227)
Supplement: S9 Table — (DOCX) [file pone.0335227.s009.docx]

**S9 Table.**

| **SNP Type** | **Minor Allele**  **Frequency** | **Number of SNPs in Korean soybean core collection** | |
| --- | --- | --- | --- |
| 19,829 rare alleles in 497 PI Soybean Collection | Monomorphic | 9,175 | 46.27% |
|  | <0.01 | 9,651 | 48.67% |
|  | 0.01~0.05 | 991 | 5.00% |
|  | 0.05~0.10 | 10 | 0.05% |
|  | 0.10~0.20 | 0 | 0.00% |
|  | 0.20~0.30 | 0 | 0.00% |
|  | 0.30~0.40 | 1 | 0.01% |
|  | 0.40> | 1 | 0.01% |
|  | **Total** | 19,829 |  |
